# Effect of Trace Mineral and Vitamin Injections on Performance, Immunity, Mineral Status, and Antioxidant Responses of Nellore Calves During the Pre-Weaning Phase

**DOI:** 10.3390/ani16030473

**Published:** 2026-02-03

**Authors:** Ronaldo G. Silva Júnior, Luciana N. Rennó, Matheus F. L. Ferreira, Ceres M. Della Lucia, Cristina M. Veloso, Aline G. da Silva, Naiara A. Marcos, Sidnei A. Lopes, Mateus A. Gonçalves, Lívya A. Oliveira, Gustavo C. M. M. Muanis

**Affiliations:** 1 Departament of Animal Science, Universidade Federal de Viçosa, Viçosa 36570-900, MG, Brazil; lucianarenno@ufv.br (L.N.R.); cristina.veloso@ufv.br (C.M.V.); naiara.marcos@ufv.br (N.A.M.); sidnei.lopes@ufv.br (S.A.L.); mateus.a.goncalves@ufv.br (M.A.G.); gustavomabub.vet@hotmail.com (G.C.M.M.M.); 2 Hill Farm Research Station, Louisiana State University, Homer, LA 71040, USA; mferreira@agcenter.lsu.edu; 3 Departament of Nutrition and Health, Universidade Federal de Viçosa, Viçosa 36570-900, MG, Brazil; cmdellalucia@ufv.br (C.M.D.L.); livya.oliveira@ufv.br (L.A.O.); 4 College of Veterinary Medicine and Animal Science, Universidade Federal do Mato Grosso do Sul, Campo Grande 79070-900, MS, Brazil; aline.g.silva@ufms.br

**Keywords:** antioxidant status, beef calves, injectable supplementation, immune response, metabolic profile

## Abstract

The objective of this research was to investigate how injectable trace minerals and vitamins influence the mineral and vitamin status, hematological parameters, antioxidant capacity, metabolic profile, and performance of Nellore calves during the pre-weaning phase. A total of 38 grazing Nellore male calves with access to creep feeding were randomly assigned to two treatments: injectable supplementation of saline solution (saline; *n* = 19) or injectable supplementation of trace minerals (copper, manganese, zinc, and selenium) and vitamins (A and E) (ITMV; *n* = 19), administered on days 75 and 150 of the calves’ age. The injectable supplementation did not affect (*p* > 0.05) body weight, average daily gain, body measurements, or carcass measurements throughout the experimental period from 75 to 218 days (7 days before weaning) of age. However, ITMV-treated calves showed higher (*p* ≤ 0.05) liver concentrations of Cu and Se on day 195 than saline calves. Higher plasma concentrations of Cu (days 195 and 218) and Se (days 135, 195, and 218) were observed for ITMV calves compared to the saline group. Furthermore, the ITMV group showed greater (*p* ≤ 0.05) glutathione peroxidase activity, higher red blood cell count, hemoglobin, and hematocrit levels, and a lower neutrophil/lymphocyte ratio (*p* = 0.04). In the metabolic profile, only a day effect was observed (*p* ≤ 0.05). In conclusion, the injectable supplementation of trace minerals and vitamins in suckling Nellore calves improved copper and selenium status and optimized antioxidant capacity and hematological parameters, in addition to modulating the immune response towards a less inflammatory state, although it did not affect performance or the metabolic profile.

## Simple Summary

This research showed that giving trace mineral and vitamin injections to nursing Nellore calves significantly benefits their health. The treatment increased the levels of key nutrients, such as the minerals copper and selenium, in the blood and liver, which helped the animals’ bodies defend themselves more effectively. In practice, these nutrients helped balance the body’s defenses, directing the immune response towards a less inflammatory state. On the other hand, the study found that these health improvements did not lead to extra weight gain or faster growth. This suggests that this type of supplement acts more as a protector of the immune system than as a tool to speed up the physical growth of the animals.

## 1. Introduction

Supplementation in the pre-weaning period (creep feeding) is a well-established strategy to maximize weight gain in beef calves, as demonstrated by a meta-analysis conducted by Carvalho et al. [1]. However, the productive success of this period depends not only on growth but also on building a robust physiological foundation that enables the calf to face subsequent immunological challenges. The occurrence of neonatal diseases and high vulnerability during the low-immunity phase [2] demonstrate that providing a supplement through a creep-feeding system aimed solely at growth may be insufficient to optimize the calf’s immune system and antioxidant defense [3]. Strengthening the antioxidant defense and the immune system during this phase is crucial, as suckling calves face intense oxidative imbalance. This challenge is generated by normal physiological processes, such as the high metabolic demand for growth and the inflammatory response itself to combat pathogens, which are the main sources of free radicals in ruminants [4,5]. The scenario becomes even more critical in young animals, whose endogenous antioxidant defense system is still maturing, a factor that increases susceptibility to diseases [6].

This physiological foundation is fundamental: a robust immune system and the ability to combat oxidative stress are critical components of a calf’s resilience [7]. These processes are governed by a complex interaction between vitamins and trace minerals. Vitamin A stands out among vitamins for its crucial role in maintaining the integrity of mucous membranes, which function as the first barrier against pathogens [8,9]. Vitamin E, the main fat-soluble antioxidant, acts in synergy with selenium (Se) to protect cell membranes from oxidative damage, while selenium is the central component of the enzyme glutathione peroxidase, which works to reduce oxidative stress [10]. Additionally, copper (Cu) is essential for the maturation of defense cells and for iron metabolism [7]. Deficiency of these nutrients, common in grazing systems [11], can result in animals with lower physiological resilience.

However, ensuring an adequate supply of these essential nutrients through conventional methods presents significant challenges. Oral supplementation of trace minerals, despite its benefits, has limitations, such as the high variability in voluntary intake among animals [12,13] and the reduced bioavailability of minerals due to antagonists present in the diet [7,10]. Given this, injectable micromineral and vitamin supplementation emerges as a strategic solution designed to complement this supplementation system. By ensuring the delivery of a precise and highly bioavailable dose of key nutrients, this technology aims to directly modulate the animal’s health, strengthening its antioxidant defense and immune system [7]. This approach follows the current trend of early-life priming, where systemic intervention during the nursing phase aims to optimize the physiological set point of the calf before the acute stress of weaning. Despite this potential, a significant knowledge gap remains, as research on injectable minerals and vitamins (ITMV) focuses almost exclusively on weaning [14,15,16] rather than proactive modulation during the pre-weaning.

In this context, it is hypothesized that injectable supplementation, even in calves with adequate nutritional support, acts as a health modulator, enhancing the antioxidant status and immune system, regardless of its effects on productive performance. Therefore, the objective of this study was to evaluate the effects of the injectable supplementation of copper, manganese, zinc, selenium, and vitamins A and E on the mineral and vitamin status, hematological parameters, antioxidant capacity, the metabolic profile, and performance of Nellore calves during the pre-weaning phase.

## 2. Materials and Methods

The study was conducted at the teaching, research, and extension unit for beef cattle (20°46′27.9″ S 42°51′45.9″ W) of the Department of Animal Science at the Universidade Federal de Viçosa (UFV), Viçosa, MG, Brazil, and approved by the UFV Ethics Committee on the Use of Production Animals (129/2023).

### 2.1. Experimental Design and Calf Management

Thirty-eight male Nellore calves [body weight (BW) = 120.5 ± 13.5 kg; 2.5 mo of age] born from Nellore cows were used in the study from 1 January to 30 May. Calves were born in eight *Urochloa decumbens* pastures (averaging 5.3 ha each), and 14 days prior to the start of the experiment, cow–calf pairs were randomly re-assigned among these pastures (4 or 5 cow–calf pairs per pasture) for environmental adaptation. At birth, all calves were identified, weighed, and subjected to standard management protocols, including navel disinfection. The cow–calf pairs were kept in the pastures with free access to water and mineral supplementation. On d 75 (75 days of age), the calves were randomly assigned into 1 of 2 treatments: (1) saline—saline injection (0.9% NaCl); or (2) injection of trace minerals and vitamins (ITMV). The ITMV solution composition consisted of 10 mg Cu/mL, 10 mg Mn/mL, 40 mg Zn/mL, 5 mg Se/mL, 3.5% vitamin A palmitate, and 5% vitamin E acetate (Kit Adaptador, Min e Vit, Biogénesis Bagó, Curitiba, Brazil). To control for the injection procedure and handling stress, both the saline solution and ITMV were administered subcutaneously in two doses of 1 mL/50 kg of BW, with one injection on each side of the neck. This standardized approach was maintained across both groups, as the ITMV protocol required minerals and vitamins to be administered separately. The treatments were applied on d 75 and 150. To minimize the influence of grazing on the calves’ mineral dynamics, both treatments were allocated to each pasture.

Starting on day 75, all calves received an energy-protein supplement fed through a creep-feeding system at 7.6 g/kg BW [1] and containing 22% crude protein (Table 1) and 3% mineral mix (dicalcium phosphate (50%), common salt (43.24%), sulfur flower (3.3%), magnesium oxide (1.6%), zinc sulfate (0.85%), copper sulfate (0.7%), manganese oxide (0.2%), cobalt sulfate (0.05%), potassium iodate (0.05%), and sodium selenite (0.01%)). The amount of feed supplement was adjusted every 30 days according to the calves’ BW. The trial period lasted from 75 to 218 days (7 days before weaning) of calf age.

Before starting the experiment, the calves were tested for Bovine Viral Diarrhea Virus (BVDV) (IDEXX BVDV Ag Point-of-Care Test, IDEXX Brasil Laboratórios Ltda., São Paulo, Brazil), to detect Persistently Infected (PI) calves; however, no PI animals were detected.

### 2.2. Data and Sample Collection

#### 2.2.1. Pasture Samples

Every 30 days, pasture was collected to quantify the forage mass by cutting close to the ground (5 cm) in five areas delimited by a 0.5 × 0.5 m square, randomly selected in each experimental paddock. Along with the pasture collection, a hand sample of the forage in each paddock was taken to assess the quality of the pasture. The samples were then dried in a ventilated oven at 55 °C for three days and ground to 1 mm for later chemical and mineral composition analysis. The average forage dry matter (DM) availability was as follows: d75 = 3.60 t/ha; d105 = 3.70 t/ha; d135 = 4.00 t/ha; d165 = 4.30 t/ha; and d195 = 4.70 t/ha. The chemical composition of the forage and supplement according to experimental days are presented in Table 1.

#### 2.2.2. Body Weight and Body Measurements

Calf full BW was collected on d 75, 105, 136, 165, 195, and 218 (7 days before weaning) of study. Body measurements were recorded with a Hypometer and flexible tape measure on days 75 and 218 (7 days before weaning), namely: height at the withers (distance from the highest point of the scapula to the ground), body length (distance between the lower tip of the scapula and the tip of the ischium), and thoracic perimeter (body circumference immediately posterior to the forelegs).

#### 2.2.3. In Vivo Carcass Traits

Loin eye area (LEA) and dorsal subcutaneous fat thickness (SFT) were measured on days 75 and 218 (7 days before weaning). Ultrasound images were taken between the 12th and 13th ribs in a transverse direction to the longissimus dorsi muscle. Fat thickness was measured in the distal middle third of the loin eye area. In addition, other images were taken in the croup region, between the ilium and ischium bony protuberances, to measure rump fat thickness (RFT). The ultrasound machine used was an Aloka model (SSD 500V, Aloka Co., Ltd., Tokyo, Japan), with a 17.2 cm linear transducer and a frequency of 3.5 MHz. LEA, SFT, and RFT were measured on the image generated by the ultrasound, using the equipment’s operating tools. The images were analyzed using the BioSoft Toolbox 2.0 for Beef software (Biotronics Inc., Ames, IA, USA).

#### 2.2.4. Blood Samples

Blood samples were collected on d 75, 135, 195, and 218 (7 days before weaning) by jugular vein puncture, using a vacuum tube with clot activator and serum separator gel (9 mL; BD SST II Advance, São Paulo, Brazil) to quantify total proteins, albumin, urea, insulin-like growth factor type 1 (IGF-1), and vitamins (A and E); the tube containing sodium fluoride with EDTA (4 mL; BD Vacutainer^®^ Fluoride/EDTA, São Paulo, Brazil) was used for glucose analysis; and the tube with sodium heparin (6 mL; BD Vacutainer^®^ Plus, São Paulo, Brazil) was used to quantify trace minerals (Cu, Zn, Se, and Mn). To quantify the antioxidant enzymes (glutathione peroxidase and superoxide dismutase), blood was collected on days 195 and 218 (7 days before weaning) in a tube containing sodium heparin (6 mL; BD Vacutainer^®^ Plus, São Paulo, Brazil). To quantify the complete blood count with differential leukocytes, the blood count was collected on day 218 (7 days before weaning) in a tube containing EDTA (BD Vacutainer EDTA, São Paulo, Brazil).

After collection, the blood samples were immediately stored on ice and then centrifuged at 2200× *g* for 20 min at 4 °C, and then the serum or plasma was packed in microtubes, identified, and stored at −20 °C for further analysis. The heparinized blood samples for analyzing antioxidant enzymes were processed according to the manufacturer’s recommendations. The tubes containing EDTA for blood count analysis were used on whole blood.

#### 2.2.5. Liver Samples

Liver samples were collected on days 75 and 195 of the experimental period (12 calves/treatment). Briefly, liver samples were collected between the 11th and 12th intercostal space of the right rib cage, using a PICKUP model bone marrow biopsy needle (PJT1115; 11-gauge × 15 cm, São Paulo, Brazil). After collection, the samples were immediately frozen at −20 °C and sent to a laboratory for mineral analysis.

#### 2.2.6. Milk Samples

To estimate the quantity and composition of milk consumed by the calves, cows were milked on day 170 of the experiment. Milking procedures were performed as described by Boggs et al. [18], with a controlled suckling period before calf separation. To empty their udders, the calves were separated from their mothers from 3 pm to 5.45 pm, when they were reunited with their mothers and allowed to suckle. At 6 pm, the calves were separated from their mothers again until the following morning. Milking was carried out at 06:00 the following day after injecting 1 ml of oxytocin (10 UI/mL; Ocitovet^®^, Paulínia, Brazil) into the cow’s mammary vein to ensure milk let-down. Unlike dairy breeds, Nellore cows often retain milk under experimental handling due to their strong maternal bond and high sensitivity to stress. Finally, the milk produced was weighed. The exact time that each cow started milking was recorded. Daily milk production was calculated as described by Lopes et al. [19]. A 30 mL sample of milk was collected from each cow to evaluate the composition and stored at 4 °C, being analyzed immediately after collection.

### 2.3. Laboratory Analysis

#### 2.3.1. Hormone and Metabolites

Blood concentrations of total protein (colorimetric test, Bioclin K031), albumin (bromecresol green method, Bioclin K040), glucose (enzymatic glucose oxidase–peroxidase method, Bioclin K082), urea (fixed-time kinetic method, Bioclin K056), glutathione peroxidase activity (GSH-px; enzymatic method, Randox RS504), and superoxide dismutase activity (SOD; colorimetric method, Randox SD125) were quantified using an automated biochemical analyzer (Mindray, BS200E, Shenzhen, China). IGF-1 concentrations were quantified using Siemens kits (Berlin, Germany) on an automated chemiluminescence analyzer (Immulite 2000, Siemens-Healthcare GmbH, Erlangen, Germany). Globulins were calculated by the difference between total proteins and albumin. Serum urea nitrogen (SUN) was estimated at 46.67% of total serum urea.

#### 2.3.2. Hematology

Red blood cell (RBC) and white blood cell (WBC) count and hemoglobin (HB) concentrations were quantified using the Hematoclin 2.8 Vet automatic analyzer (Bioclin, Belo Horizonte, Brazil), the differentiation of leukocyte cells (neutrophils and lymphocytes) was determined under a microscope, and neutrophil to lymphocyte (N:L) ratios were calculated. Hematocrit (HTC) was quantified in a microcentrifuge (MicroSpin microhematocrit centrifuge, Jaboticabal, Brazil).

#### 2.3.3. Mineral and Vitamins

Plasma samples were digested in sealed vials in a microwave oven (DGT-100 PLUS, Provecto Analítica, Campinas, Brazil), and readings for quantification of Cu and Zn concentrations were performed by inductively coupled plasma atomic emission spectrometry (ICAP 6000 Series—DUO, Thermo Scientific, Campinas, Brazil), and Mn and Se readings were performed by graphite furnace atomic emission spectrometry (ICE 3000 Series, Thermo Scientific, Campinas, Brazil) in a commercial laboratory (Bio Minerais Análises Químicas Ltda., Campinas, Brazil).

Liver samples were digested in sealed vials in a microwave oven (DGT-100 PLUS, Provecto Analítica, Campinas, Brazil), and readings for quantification of Cu, Zn, and Mn concentrations were performed by inductively coupled plasma atomic emission spectrometry (ICAP 6000 Series—DUO, Thermo Scientific, Campinas, Brazil), and the Se reading was performed by atomic emission spectrometry in a graphite furnace (ICE 3000 Series, Thermo Scientific, Campinas, Brazil) in a commercial laboratory (Bio Minerais Análises Químicas Ltda., Campinas, Brazil).

The concentrations of vitamins A and E in plasma were determined by High-Performance Liquid Chromatography (HPLC; SCL-10A VP, Shimadzu Scientific Instruments Inc., Kyoto, Japan), as described by Turner and Burri [20] in the Vitamin Analysis Laboratory of the Department of Nutrition and Health at the Federal University of Viçosa. The analyses were performed on an HPLC system equipped with a Diode Array Detector (DAD; SOD-M10 AVP, Shimadzu Scientific Instruments Inc., Kyoto, Japan). Chromatographic separation was achieved using a Phenomenex Gemini reverse-phase C18 column (250 mm × 4.6 mm, 5 µm) coupled with a Phenomenex ODS C18 guard column (4 mm × 3 mm). The isocratic mobile phase consisted of acetonitrile, dichloromethane, and methanol (70:20:10, *v*/*v*/*v*) at a flow rate of 1.0 mL/min. Simultaneous detection was performed at 292 nm (tocopherols) and 325 nm (retinol), with a total run time of 24 min.

#### 2.3.4. Chemical Composition of Pasture and Supplement

The forage and supplement samples were analyzed according to the Brazilian National Institute of Science and Technology in Animal Science (INCT-CA) [17] for dry matter (DM; method G-003/1), crude protein (CP; method N-001/2), ash (method M-001/2), and neutral detergent fiber (NDF; method F-002/2). The forage and supplement samples were digested in sealed vials in a microwave oven (DGT-100 PLUS, Provecto Analítica, Campinas, Brazil), and the readings for quantifying the concentrations of Cu, Zn, and Mn in these samples were performed by inductively coupled plasma atomic emission spectrometry (ICAP 6000 Series—DUO, Thermo Scientific, Campinas, Brazil), and Se readings were performed by atomic emission spectrometry in a graphite furnace (ICE 3000 Series, Thermo Scientific, Campinas, Brazil) in a commercial laboratory (Bio Minerais Análises Químicas Ltda., Campinas, Brazil). The concentrations of beta-carotene and vitamin E in the forage and supplement samples were quantified at the Vitamin Analysis Laboratory of the Department of Nutrition and Health at the Federal University of Viçosa. Quantification was performed by High-Performance Liquid Chromatography (HPLC; SCL-10A VP, Shimadzu Scientific Instruments Inc., Kyoto, Japan), following the methodology of Turner and Burri [20]. The HPLC system was equipped with a Diode Array Detector (DAD; SOD-M10 AVP, Shimadzu Scientific Instruments Inc., Kyoto, Japan). The quantification of vitamin E was performed using High-Performance Liquid Chromatography with a Phenomenex Luna Silica (2) normal-phase column (250 mm × 4.6 mm, 5 µm, 100 Å), protected by a normal-phase Silica guard column (4.0 mm × 3.0 mm, Phenomenex). The mobile phase consisted of hexane, isopropanol, and glacial acetic acid (98.9:0.6:0.5, *v*/*v*/*v*), delivered at a flow rate of 1.0 mL/min. Detection was carried out at 450 nm, with a total run time of approximately 16–22 min. For β-carotene determination, a Shimadzu High-Performance Liquid Chromatography system equipped with a UV-visible Diode Array Detector was used, operating at 450 nm. The mobile phase was composed of methanol, ethyl acetate, and acetonitrile (80:10:10, *v*/*v*/*v*), at a flow rate of 2.0 mL/min. Separations were performed on a reverse-phase RP-18 column (Phenomenex Gemini C18, 5 µm, 250 mm × 4.6 mm), equipped with a Phenomenex ODS (C18) guard column (4 mm × 3 mm), with a run time of approximately 12–15 min.

#### 2.3.5. Milk Composition

The fresh milk samples were analyzed for protein, fat, lactose, and total solids using an ultrasonic analyzer (Lactoscan SP, Milkotronic LTD, Nova Zagora, Bulgaria).

### 2.4. Statistical Analysis

The basic statistical model used was as follows:

Yijk = μ + Pi + Cj + e(ij)k

where Yijk = observation made on animal k, belonging to treatment j, in paddock i; μ = general constant; Pi = effect of paddock I (random); Cj = effect of treatment (saline or ITMV) j (fixed); and e(ij)k = random effect, not observable, and considered NIID (0, σ2e).

Data were analyzed in a completely randomized design, considering the calf as the experimental unit for all analyses. All variables were analyzed using the MIXED procedure in SAS 9.4 (Inst. Inc., Cary, NC, USA). The variables of performance, milk production, mineral and vitamin status, hematology, and oxidative status were tested for fixed effects of treatment, while the variables of metabolic profile were analyzed as repeated measures and tested for fixed effects of treatment, day, and treatment × day, where the best structure of the (co)variance matrix was chosen based on the corrected Akaike information criterion. The initial results were included as covariates in each respective variable but were removed from the model when *p* > 0.05. Means were separated using Pairwise Difference (PDIFF), and all results were reported as LSMEANS followed by SEM. Significance was defined as *p* < 0.05.

## 3. Results

### 3.1. Performance, Milk Production, and Metabolic Profile

No effects of ITMV were observed for the dam’s milk yield or milk composition (*p* ≥ 0.98; Table 2). No effects of ITMV were observed for BW (*p* ≥ 0.13), average daily gain (*p* ≥ 0.60), body measurements (*p* ≥ 0.18), and carcass measurements (*p* ≥ 0.24; Table 3).

An effect of the day (*p* < 0.0001) but no effect of ITMV (*p* ≥ 0.34) or an interaction of the ITMV × day (*p* ≥ 0.16) were observed for blood concentrations of glucose, total proteins, albumin, globulins, SUN, and IGF-1 (Table 4).

**Figure 1:**
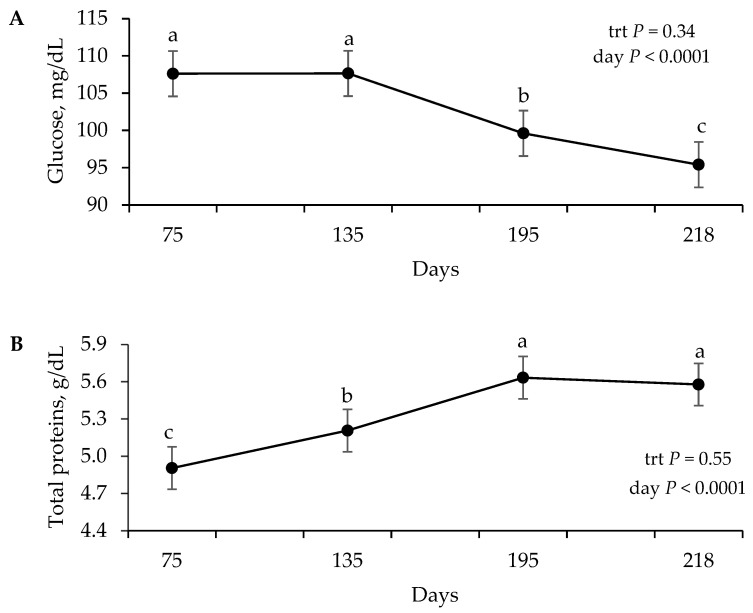

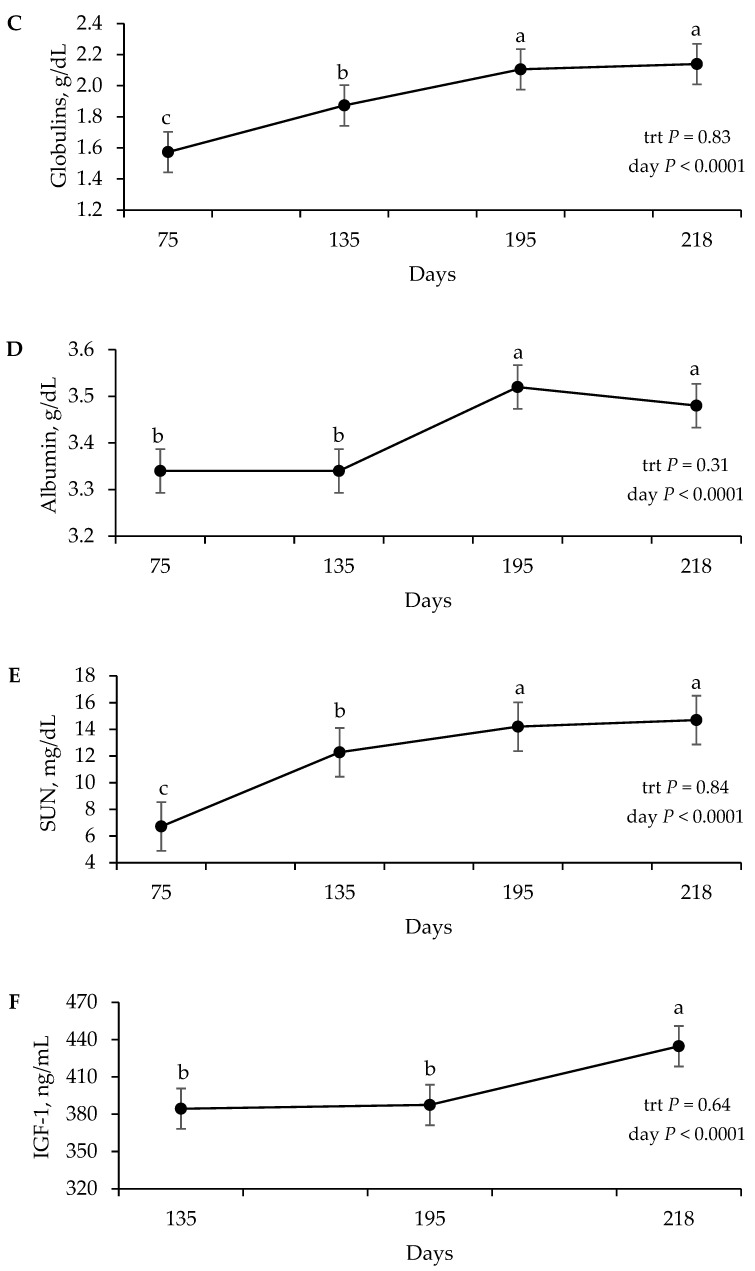
Glucose (**A**), total protein (**B**), globulin (**C**), albumin (**D**), serum urea nitrogen (SUN) (**E**), insulin-like growth factor type 1 (IGF-1) (**F**), and serum concentrations of Nellore calves receiving saline solution (saline; *n* = 19) or injection of trace minerals and vitamins (ITMV; *n* = 19) during the pre-weaning phase. Means with different superscript letters differ between days (*p* < 0.05). trt: treatment effect; day: day effect.

Plasma glucose concentrations decreased (*p* < 0.0001) throughout the experimental period (Figure 1A). The values on days 75 and 135 were similar to each other and higher than on subsequent days. A significant reduction was observed on day 195, with the lowest concentration recorded at the end of the period on day 218. Serum total proteins increased (*p* < 0.0001) from day 75 to day 195, remaining high and stable until day 218 (Figure 1B). This increase was driven mainly by globulins, which followed the same pattern as the total proteins (*p* < 0.0001; Figure 1C). For albumin, lower values were observed on days 75 and 135, followed by an increase on days 195 and 218, which were similar (*p* < 0.0001; Figure 1D). Similarly to total proteins, SUN levels increased (*p* < 0.0001) as the calves aged (Figure 1E). The lowest value was observed at the first collection (day 75), with subsequent increases until days 195 and 218, which were the highest and did not differ from each other. The concentration of IGF-1, evaluated from day 135 onwards, was also affected by time (*p* < 0.0001; Figure 1F). On days 135 and 195, the values were lower, and subsequently, a sharp increase was observed, with the peak concentration being reached on day 218.

### 3.2. Mineral and Vitamin Status

There were no effects of ITMV (*p* ≥ 0.21) on liver concentrations of Cu, Mn, Zn, and Se before the start of supplementation (75 days of age). However, ITMV calves had higher liver concentrations of Cu and Se on day 195 of age compared to saline calves (*p* = 0.03). Yet there was no effect of ITMV (*p* ≥ 0.48) on the concentrations of Mn and Zn on the same day (Table 5).

No effects of ITMV were observed on plasma concentrations of Mn, Zn, and vitamin A on days 75, 135, 195, and 218 or on Cu concentrations on days 75 and 135 (*p* ≥ 0.10). However, ITMV calves had higher plasma concentrations of Cu on days 195 and 218 compared to saline calves (*p* = 0.01; Table 6). There was an effect of ITMV (*p* = 0.02) on plasma concentrations of Se on days 135, 195, and 218 (Table 6). Plasma concentrations of vitamin E were detected in all samples but were below the quantification limit of the analytical method.

### 3.3. Oxidative Status and Hematology

There was an effect of ITMV on glutathione peroxidase concentrations on days 195 and 218 (*p* = 0.02), where ITMV calves had higher concentrations of this antioxidant enzyme compared to saline calves (Table 7). However, there was no effect of ITMV on the superoxide dismutase concentration on the same days (*p* ≥ 0.83; Table 7).

Effect of ITMV was observed on red blood cells (*p* = 0.04), hemoglobin (*p* = 0.04), hematocrit (*p* = 0.03), and the N:L ratio (*p* = 0.04), where ITMV calves showed higher concentrations of these blood cells and a lower N:L ratio compared to saline calves (Table 7). However, no effect of ITMV was found on white blood cells, lymphocytes, neutrophils, monocytes, eosinophils, and platelets (*p* ≥ 0.34; Table 7).

## 4. Discussion

The lack of effects from the injectable supplementation on performance and blood metabolites is consistent with the animals’ high plane of nutrition, supported by access to creep feeding and adequate milk production from the dams. This condition was evidenced by high concentrations of IGF-1, high performance, and high average daily gain, which likely masked potential performance benefits of the supplementation injectable. Some studies that used injectable trace mineral supplementation during the pre-weaning and weaning phases found no effects on calf performance [14,15,16], which is consistent with the results of the present study. However, other studies have reported greater performance in animals supplemented with injectable trace minerals or with injectable trace minerals and vitamins only at weaning [21,22] and not in calves in the pre-weaning phase. The growth response of animals to ITMV supplementation is influenced by a series of variables, such as the magnitude of stress, breed, and, above all, their nutritional (mineral) status. The complex interaction among multiple factors justifies the occurrence of conflicting results in research on this topic.

The metabolism results demonstrate a state of intense anabolism, evidenced by the increase in IGF-1, which is consistent with the endocrine and metabolic responses observed in Nellore heifers subjected to different supplementation levels [23]. The increase in serum proteins reflects an improvement in protein metabolism, serving as important indicators in the metabolic profile of herds [24]. Concomitantly, the increase in serum urea nitrogen reflects rumen development and the calf’s transition into a functional ruminant, during which solid feed intake and nitrogen metabolism become more intense [25]. This profile is consistent with the observations of Saraiva [26] on calves under high supplementation.

Injectable supplementation is an efficient strategy to increase the body stores of trace minerals, specifically copper and selenium [27]. The liver is the organ where most trace minerals of interest for cattle are stored, incorporated into enzymes, and released when needed. The collection of this tissue is described as a reference method and the best way to determine the concentrations and nutritional status of trace minerals in cattle [7]. The increase in liver trace mineral concentrations after the application of an injectable trace mineral (ITM) confirms the effectiveness of the absorption and storage process. This finding is consistent with other experiments described in the literature; Arthington et al. [14] also reported an increase in hepatic Cu and Se status in ITM calves before weaning, while Vedovatto et al. [16] reported similar results at weaning. Similarly, Genther-Schroeder et al. [28] observed the same increase in ITM steers during preconditioning, 28 days before transport. Other studies, such as that by Pogge et al. [29], also confirmed the hepatic increase in Cu and Se in steers of different breeds in feedlots. Ensuring adequate hepatic levels before periods of intense stress, such as weaning and transport, is essential, since the demand for these minerals to support immune and antioxidant responses increases precisely during these challenging times.

In the present study, plasma concentrations of Cu and Se remained within the physiological ranges considered adequate for beef calves [30]. The saline group presented mean values of 638 µg/L for Cu and 54 µg/L for Se, while the IMV group showed 685 µg/L and 66 µg/L, respectively. Arthington and Havenga [31] obtained serum concentrations for beef calves aged 10 to 12 months of 720 µg/L for Cu and 94 µg/L for Se, maintaining or increasing these levels following injectable supplementation. According to the thresholds reviewed by Spears et al. [30], plasma concentrations above 600 µg/L for Cu and 30 µg/L for Se indicate an adequate nutritional status, while levels below 400 µg/L and 25 µg/L are considered deficient, respectively. Therefore, these results indicate that ITMV supplementation optimized circulating levels of Cu and Se, even in calves maintaining an adequate physiological status of these minerals.

According to Daniel and Martín-Tereso [32], the main homeostatic adaptation of ruminants to variable mineral supply is the regulation of apparent absorption efficiency, including excretion, as demonstrated by Abrams et al. [33], who observed a 12-fold increase in the biliary excretion rate of the injected mineral (^54^Mn) in calves pre-fed a Mn-rich diet. Similarly, zinc homeostasis is maintained by regulating fecal excretion. Miller et al. [34] proved this mechanism by injecting ^65^Zn into ruminants, showing that calves fed a normal diet (46 ppm Zn) excreted a higher percentage of the injected ^65^Zn in their feces. The lack of effect on Zn and Mn levels does not indicate the ineffectiveness of injectable supplementation but rather that the animals probably already had an adequate nutritional status for these microminerals supplied by the basal diet, possibly leading to the activation of specific and highly efficient post-absorptive homeostatic mechanisms for each mineral.

The plasma concentrations of vitamin A also did not vary at any of the evaluated time points. This is likely because maternal milk is rich in vitamin A, and forages are generally rich sources of β-carotene, the precursor to vitamin A [35,36], which contributes to the vitamin status of the calves. In animals with adequate liver reserves of vitamin A, additional supplementation may not cause significant changes in circulating levels [37].

An increase in the concentration of GSH-px following ITMV application has also been observed in other studies [15,16,22]. The improved status of Cu and Se resulted in direct and beneficial physiological effects, which increase the efficiency of the body’s defense systems. Selenium acts as an essential cofactor for several enzymes, which highlights its role as a structural component of GSH-px, one of the most potent antioxidants found in mammals [12]. The primary function of this enzyme is to protect cells from oxidative stress by catalyzing the reduction in hydroperoxides, which includes converting hydrogen peroxide into water and neutralizing lipid hydroperoxides [7,38]. Since the injected Se is necessary for the synthesis of this enzyme, it is predictable that its concentration would increase in animals receiving an injectable supplementation of trace minerals and vitamins.

The activity of the enzyme SOD did not differ between treatments, a result that can be attributed to the fact that Mn status also showed no difference. This connection is biologically important, as the ability of Mn to change its valence in the mitochondrial isoform of the enzyme (MnSOD) makes it the main defender against intracellular oxidative stress. Although other SODs depend on Cu for their redox activity, MnSOD is by far the most dominant dismutase in most tissues [10].

Cu is an essential mineral for erythropoiesis (the formation of red blood cells) in cattle, as it plays a fundamental role in iron metabolism, heme and hemoglobin biosynthesis, and the mitochondrial function of precursor cells. Consequently, copper deficiency impairs iron mobilization and can lead to anemia, typically normocytic and hypochromic, with a reduction in hemoglobin and hematocrit levels [39,40]. However, trace mineral supplementation proves to be effective at restoring erythropoiesis by increasing serum Cu, red blood cells, hemoglobin, and hematocrit levels [39,41], which corroborates the findings of this study.

Complementary to the structural role of Cu in erythropoiesis, selenium plays a vital role in the protection and survival of circulating erythrocytes. Its importance lies in its function as an essential component of cytosolic glutathione peroxidase (GPX1), the main selenoprotein found in red blood cells and the defender of the erythrocyte membrane against oxidative damage. By neutralizing reactive oxygen species, the activity of GPX1 prevents premature hemolysis, ensuring the longevity and functionality of erythrocytes. Selenium deficiency, by compromising this antioxidant protection, has been associated with hemolytic anemia in ruminants, which highlights the importance of this trace mineral for maintaining adequate hematocrit and hemoglobin levels [10].

In the present study, a reduction in the neutrophil–lymphocyte ratio was observed in the calves that received the injectable supplementation. Although frequently used as a stress marker, the neutrophil–lymphocyte ratio is, fundamentally, an indicator of the balance between the innate and adaptive immune responses, serving as a valid index of systemic inflammation [42]. A lower neutrophil–lymphocyte ratio, as observed in the group that received injectable supplementation, suggests modulation towards a less inflammatory state and greater immune system homeostasis. This finding is strongly supported by the improvement in the animals’ antioxidant status. The higher activity of the selenoenzyme glutathione peroxidase in the group supplemented with trace minerals and vitamins indicates a greater capacity to neutralize oxidative stress [14], a precursor to systemic inflammation [4,5]. Therefore, it is likely that the supplementation reduced oxidative damage, decreasing the need for a chronic inflammatory response. This, consequently, resulted in a lower proportion of circulating neutrophils relative to lymphocytes, indicating a more efficient immune system, potentially better prepared to respond appropriately to health challenges rather than maintaining a high basal inflammatory response [43].

The findings of this study elucidate that, in high-performance creep-feeding systems, mineral and vitamin supplementation did not act as a performance enhancer but rather as physiological support. Optimizing the status of essential trace minerals (Cu, Se) was crucial for strengthening the antioxidant defense system and promoting immune system homeostasis. Therefore, injectable supplementation acted as a metabolic and immunity modulator, giving calves greater resilience to cope with periods of intense stress, such as weaning and transport.

## 5. Conclusions

Injectable micromineral and vitamin supplementation in nursing Nellore calves in a creep-feeding system did not affect the performance and metabolic profile; however, it improved Cu and Se status and optimized antioxidant capacity and hematological parameters, in addition to modulating the immune response towards a less inflammatory state. From a practical standpoint, injectable microminerals and vitamins serve as strategic interventions to bolster the animal’s physiological defenses, with their use being recommended as a preventive health measure in anticipation of critical management events, such as weaning or transport, when calves are most susceptible to oxidative stress and immunosuppression.

## Figures and Tables

**Table 1:** Average chemical composition and forage mass of *Urochloa decumbens* pastures according to experimental days, and chemical composition of energy-protein supplement.

Items	Supplement ^2^	Day of the Study ^1^				
		75	105	135	165	195
Forage mass, kg DM/ha	-	3600	3700	4000	4300	4700
Dry matter, g/kg	892	209	209	227	231	318
Organic matter, g/kg	950	923	912	912	917	920
Crude protein, g/kg	214	90.0	109	91.2	83.3	74.9
Neutral detergent fiber, g/kg	186	650	656	695	652	748
Cu, mg/kg	56.2	3.20	2.80	2.40	2.50	2.7
Mn, mg/kg	52.7	76.1	73.7	71.2	86.2	101
Zn, mg/kg	117	19.8	17.5	15.1	18.1	21.0
Se, µg/kg	1440	207	167	126	176	225
β-carotene, µg/100 g	32.6	1476	1098	937	908	879
Vitamin E, µg/100 g	370	2958	2827	2696	2739	2781

^1^ Forage samples were collected every 30 days. The forage and supplement samples were analyzed according to Detmann et al. [17]. DM = dry matter. ^2^ Supplement composition (as-fed basis): corn meal (57%), soybean meal (40%), and mineral mix (3%).

**Table 2:** Milk yield and composition of Nellore cows whose calves received saline solution (saline; *n* = 19) or injection of trace minerals and vitamins (ITMV; *n* = 19) during the pre-weaning phase.

Items	Treatments		SEM	*p*-Value
	Saline	ITMV		trt
Milk Yield, kg/d	6.66	6.65	0.362	0.98
Fat ^1^	5.57	5.53	0.250	0.90
Protein ^1^	3.28	3.28	0.037	0.98
Lactose ^1^	4.93	4.93	0.055	0.99
Total Solids ^1^	14.6	14.5	0.265	0.90

^1^ g/100 g; SEM: standard error mean; and trt: treatment effect. Milking was performed on d 170 of the study, according to Boggs et al. [18], and daily milk production was calculated as described by Lopes et al. [19].

**Table 3:** Growth performance of Nellore calves receiving saline solution (saline; *n* = 19) or injection of trace minerals and vitamins (ITMV; *n* = 19) during the pre-weaning phase.

Items	Treatments		SEM	*p*-Value ^1^
	Saline	ITMV		trt
Body weight, kg				
d75	118	124	3.000	0.13
d105	160	160	2.610	0.52
d135	198	198	5.483	0.98
d165	226	228	5.610	0.50
d195	256	257	8.291	0.61
d218	295	294	8.285	0.80
Average daily gain, kg				
d75 to 135	1.27	1.27	0.024	0.95
d135 to d218	1.15	1.14	0.023	0.60
Overall	1.20	1.19	0.017	0.66
Body measurements, cm				
iWH	101	102	0.794	0.42
fWH	121	121	0.679	0.86
iBL	88.2	89.8	1.421	0.33
fBL	112	113	1.260	0.84
iTP	111	113	1.715	0.18
fTP	166	168	3.951	0.43
In vivo carcass traits				
iLEA, cm^2^	32.3	34.2	1.223	0.28
fLEA, cm^2^	43.6	41.6	1.425	0.32
iSFT, mm	1.78	1.80	0.097	0.88
fSFT, mm	2.74	2.76	0.225	0.95
iRFT, mm	2.25	2.27	0.152	0.95
fRFT, mm	3.79	4.32	0.352	0.24

iWH: initial withers height; fWH: final withers height; iBL: initial body length; fBL: final body length; iTP: initial thoracic perimeter; fTP: final thoracic perimeter; iLEA: initial loin eye area; fLEA: final loin eye area; iSFT: initial dorsal subcutaneous fat thickness; fSFT: final dorsal subcutaneous fat thickness; iRFT: initial rump fat thickness; fRFT: final rump fat thickness; SEM: standard error mean; and trt: treatment effect. ^1^ Initial body weight (*p* < 0.0001) was included as a covariate for the body weight variable. Body measurements were performed using a Hypometer and flexible tape on days 75 and 218. Carcass traits were measured using an ultrasound Aloka model on days 75 and 218.

**Table 4:** Metabolic profile of Nellore calves receiving saline solution (saline; *n* = 19) or injection of trace minerals and vitamins (ITMV; *n* = 19) during the pre-weaning phase.

Items	Treatments		SEM	*p*-Value		
	Saline	ITMV		trt	Day	trt × Day
Glucose, mg/dL	101	104	1.917	0.34	<0.0001	0.50
Total Protein, g/dL	5.30	5.36	0.083	0.55	<0.0001	0.31
Albumin, g/dL	3.39	3.43	0.028	0.31	<0.0001	0.50
Globulins, g/dL	1.91	1.93	0.072	0.83	<0.0001	0.32
SUN, mg/dL	11.9	12.0	0.385	0.84	<0.0001	0.16
IGF-1, ng/mL	391	400	15.130	0.64	<0.0001	0.89

SUN: serum urea nitrogen; IGF-1: insulin-like growth factor type 1; SEM: standard error mean; trt: treatment effect; day: day effect; and trt × day: interaction between treatment and day. Serum and plasma samples were collected from a jugular vein on days 75, 135, 195, and 218.

**Table 5:** Liver mineral status in Nellore calves receiving saline solution (saline; *n* = 12) or injection of trace minerals and vitamins (ITMV; *n* = 12) during the pre-weaning phase.

Items	Treatments		SEM	*p*-Value
	Saline	ITMV		trt
Cu, mg/kg				
d75	44.4	34.0	4.819	0.21
d195	104	138	9.894	0.03
Mn, mg/kg				
d75	1118	1181	137.650	0.74
d195	1400	1453	76.444	0.63
Zn, mg/kg				
d75	17.7	17.5	1.777	0.93
d195	23.6	22.8	2.002	0.48
Se, µg/kg				
d75	72.5	68.6	18.634	0.88
d195	141	427	101.550	0.04

SEM: standard error mean; trt: treatment effect. Liver samples were collected between the 11th and 12th intercostal space using a PICKUP model bone marrow biopsy needle on days 75 and 195.

**Table 6:** Plasma trace minerals and vitamin A and vitamin E concentrations in Nellore calves receiving saline solution (saline; *n* = 19) or injection of trace minerals and vitamins (ITMV; *n* = 19) during the pre-weaning phase.

Items	Treatments		SEM	*p*-Value
	Saline	ITMV		trt
Cu, µg/L				
d75	672	671	53.669	0.98
d135	664	639	41.551	0.53
d195	669	755	29.557	0.01
d218	548	677	42.136	0.04
Mn, µg/L				
d75	5.80	5.83	2.219	0.99
d135	14.2	14.8	7.575	0.96
d195	5.42	7.67	2.253	0.49
d218	2.64	2.42	0.341	0.58
Zn, mg/L				
d75	0.65	0.72	0.047	0.33
d135	0.63	0.56	0.038	0.21
d195	0.56	0.58	0.036	0.76
d218	0.48	0.49	0.040	0.87
Se, µg/L				
d75	42.5	43.0	5.456	0.94
d135	55.2	69.3	4.004	0.02
d195	64.0	78.4	4.250	0.03
d218	65.8	75.3	3.090	0.04
Vitamin A, µg/mL				
d75	1.53	1.46	0.061	0.41
d135	2.18	1.75	0.238	0.17
d195	2.31	2.27	0.097	0.80
d218	2.55	2.29	0.107	0.10
Vitamin E, µg/mL	NQ ^1^	NQ ^1^	-	-

^1^ Not quantifiable; SEM: standard error mean; and trt: treatment effect. Plasma samples were collected from a jugular vein on days 75, 135, 195, and 218.

**Table 7:** Hematology and oxidative status of Nellore calves receiving saline solution (saline; *n* = 19) or injection of trace minerals and vitamins (ITMV; *n* = 19) during the pre-weaning phase.

Items	Treatments		SEM	*p*-Value
	Saline	ITMV		trt
Hematology				
Red Blood Cells, ×10^6^/µL	9.72	10.3	0.191	0.04
Hemoglobin, g/dL	11.1	11.8	0.222	0.04
Hematocrit, %	32.5	34.4	0.589	0.03
White Blood Cells, ×10^3^/µL	13.2	12.9	0.744	0.80
Lymphocytes, ×10^3^/µL	6.72	6.91	0.412	0.75
Neutrophils, ×10^3^/µL	5.36	4.93	0.325	0.35
Neutrophils–Lymphocytes	0.80	0.71	0.029	0.04
Monocyte, ×10^2^/µL	7.63	6.93	0.594	0.41
Eosinophils, ×10^2^/µL	4.02	3.86	0.677	0.86
Platelets, ×10^5^/µL	4.57	4.20	0.274	0.34
Oxidative status				
Glutathione Peroxidase, U/mL				
d195	2.05	2.44	0.120	0.02
d218	1.85	2.27	0.150	0.02
Superoxide Dismutase, U/mL				
d195	1.86	1.89	0.076	0.83
d218	1.79	1.76	0.112	0.84

SEM: standard error mean; trt: treatment effect. Plasma samples for hematology were collected from the jugular vein on day 218 and for oxidative status on days 195 and 218.

## Data Availability

The data were not deposited in an official repository. The data generated during the current study are available from the corresponding author upon reasonable request.

## Abbreviations

The following abbreviations are used in this manuscript:

ITMV	injection of trace minerals and vitamins
BW	body weight
BVDV	bovine viral diarrhea virus
PI	persistently infected
LEA	loin eye area
SFT	dorsal subcutaneous fat thickness
RFT	rump fat thickness
IGF-1	insulin-like growth factor type 1
SUN	serum urea nitrogen
RBC	red blood cell
WBC	white blood cell
HB	hemoglobin
N:L	neutrophil to lymphocyte ratio
HTC	hematocrit
HPLC	high performance liquid chromatography
DAD	diode array detector
DM	dry matter
CP	crude protein
NDF	neutral detergent fiber
ITM	injectable trace mineral
GSH-px	glutathione peroxidase
SOD	superoxide dismutase
GPX1	cytosolic glutathione peroxidase
